# Proteomic Analysis of KCNK3 Loss of Expression Identified Dysregulated Pathways in Pulmonary Vascular Cells

**DOI:** 10.3390/ijms21197400

**Published:** 2020-10-07

**Authors:** Hélène Le Ribeuz, Florent Dumont, Guillaume Ruellou, Mélanie Lambert, Thierry Balliau, Marceau Quatredeniers, Barbara Girerd, Sylvia Cohen-Kaminsky, Olaf Mercier, Stéphanie Yen-Nicolaÿ, Marc Humbert, David Montani, Véronique Capuano, Fabrice Antigny

**Affiliations:** 1 Faculté de Médecine, Université Paris-Saclay, 94270 Le Kremlin-Bicêtre, France; helene.leribeuz@aol.com (H.L.R.); melanie.lambert91@hotmail.fr (M.L.); quatredeniers.marceau@gmail.com (M.Q.); barbara.girerd19@gmail.com (B.G.); sylvia.cohen-kaminsky@universite.paris-saclay.fr (S.C.-K.); olaf.mercier@gmail.com (O.M.); marc.humbert@bct.aphp.fr (M.H.); davidmontani@gmail.com (D.M.); veronique.capuano@universite.paris-saclay.fr (V.C.); 2 INSERM UMR_S 999, Hypertension Pulmonaire: Physiopathologie et Innovation Thérapeutique, Hôpital Marie Lannelongue, 92350 Le Plessis-Robinson, France; 3 Assistance Publique - Hôpitaux de Paris (AP-HP), Service de Pneumologie et Soins Intensifs Respiratoires, Centre de Référence de l’Hypertension Pulmonaire, Hôpital Bicêtre, 94270 Le Kremlin-Bicêtre, France; 4 UMS Ingénierie et Plateformes au Service de l’Innovation Thérapeutique, Université Paris-Saclay, 92290 Châtenay-Malabry, France; florent.dumont@universite.paris-saclay.fr (F.D.); guillaume.ruellou@universite.paris-saclay.fr (G.R.); stephanie.nicolay@universite-paris-saclay.fr (S.Y.-N.); 5 PAPPSO-GQE-Le Moulon, INRAE, CNRS, AgroParisTech, Université Paris-Saclay, 91190 Gif-sur-Yvette, France; thierry.balliau@inrae.fr

**Keywords:** PAH, potassium channel, proteomic

## Abstract

The physiopathology of pulmonary arterial hypertension (PAH) is characterized by pulmonary artery smooth muscle cell (PASMC) and endothelial cell (PAEC) dysfunction, contributing to pulmonary arterial obstruction and PAH progression. KCNK3 loss of function mutations are responsible for the first channelopathy identified in PAH. Loss of KCNK3 function/expression is a hallmark of PAH. However, the molecular mechanisms involved in KCNK3 dysfunction are mostly unknown. To identify the pathological molecular mechanisms downstream of KCNK3 in human PASMCs (hPASMCs) and human PAECs (hPAECs), we used a Liquid Chromatography-Tandem Mass Spectrometry-based proteomic approach to identify the molecular pathways regulated by KCNK3. KCNK3 loss of expression was induced in control hPASMCs or hPAECs by specific siRNA targeting KCNK3. We found that the loss of KCNK3 expression in hPAECs and hPASMCs leads to 326 and 222 proteins differentially expressed, respectively. Among them, 53 proteins were common to hPAECs and hPASMCs. The specific proteome remodeling in hPAECs in absence of KCNK3 was mostly related to the activation of glycolysis, the superpathway of methionine degradation, and the mTOR signaling pathways, and to a reduction in EIF2 signaling pathways. In hPASMCs, we found an activation of the PI3K/AKT signaling pathways and a reduction in EIF2 signaling and the Purine Nucleotides De Novo Biosynthesis II and IL-8 signaling pathways. Common to hPAECs and hPASMCs, we found that the loss of KCNK3 expression leads to the activation of the NRF2-mediated oxidative stress response and a reduction in the interferon pathway. In the hPAECs and hPASMCs, we found an increased expression of HO-1 (heme oxygenase-1) and a decreased IFIT3 (interferon-induced proteins with tetratricopeptide repeats 3) (confirmed by Western blotting), allowing us to identify these axes to understand the consequences of KCNK3 dysfunction. Our experiments, based on the loss of KCNK3 expression by a specific siRNA strategy in control hPAECs and hPASMCs, allow us to identify differences in the activation of several signaling pathways, indicating the key role played by KCNK3 dysfunction in the development of PAH. Altogether, these results allow us to better understand the consequences of KCNK3 dysfunction and suggest that KCNK3 loss of expression acts in favor of the proliferation and migration of hPASMCs and promotes the metabolic shift and apoptosis resistance of hPAECs.

## 1. Introduction

*Potassium Channel Subfamily K Member 3 (KCNK3)* was identified in 2013 as a predisposing gene for pulmonary arterial hypertension (PAH) [1]. To date, 12 different mutations in *KCNK3* have been described in PAH patients [2]. PAH is a severe and progressive disease, with an estimated prevalence of 15 to 50 per million of the population [3]. Under the new clinical classification [4], PAH consists of different etiologies leading to precapillary pulmonary hypertension, which is hemodynamically proposed to be defined by a mean pulmonary artery pressure (mPAP) >20 mmHg, a pulmonary artery wedge pressure <15 mmHg, and a pulmonary vascular resistance (PVR) >3 Wood units at rest [4]. *KCNK3* mutations carrier patients have a higher mPAP and are younger than idiopathic PAH (iPAH) patients at diagnosis [2]. All the identified *KCNK3* mutations result in a loss of function in KCNK3 channels [1,2]. KCNK3, also known as Twik-related-acid-sensitive-K^+^ channel (TASK-1) or K2P3.1, displays several electrophysiological characteristics of background K^+^ current, including minimal voltage sensitivity, extracellular pH sensitivity, resistance to classical K^+^ channel inhibitors, and insensitivity to cytoplasmic Ca^2+^ [5]. TASK-1 channels are expressed in pulmonary arterial smooth muscle cells (PASMCs) and endothelial cells (PAECs) [6].

In the absence of *KCNK3* mutations, we found a reduced TASK-1 function or expression in lung or isolated pulmonary arteries (PA) from heritable PAH (*BMPR2* carriers patients) and iPAH patients, as well as in isolated PASMCs, demonstrating that reduced KCNK3 function/expression is a hallmark of PAH at the pulmonary vascular level [7]. Otherwise, we recently found that *Kcnk3* deficiency in rats (CRISPR/Cas9 technology) leads to higher susceptibility to monocrotaline- or chronic hypoxia-induced PH, confirming that KCNK3 loss of function predisposes one to the development of PAH. We also found that *KCNK3* knockdown enhances proliferation in control human PASMCs (hPASMCs) [8].

In PAH, the PA obstruction is multifactorial, involving at least hPAECs and hPASMCs dysfunctions, leading to excessive PA vasoconstriction, an over-proliferation of PASMCs and PAECs associated with apoptosis resistance, and pro-inflammatory phenotypes. Most of these phenotypes were persistent in vitro in cultured hPASMCs and hPAECs from PAH patients [9].

In pulmonary vascular cells, K^+^ channels play major role in the control of cell contraction, cell proliferation and survival, cell volume, and cell apoptosis [2]. However, the role of KCNK3 dysfunction in the proliferation/apoptosis imbalance of hPASMCs and hPAECs is still unclear, as well as the molecular mechanism link to KCNK3 dysfunction and the aberrant phenotype of hPASMCs or hPAECs in PAH.

To identify the pathological molecular mechanisms induced by KCNK3 loss of expression in hPASMCs and hPAECs, we postulated that a differential proteomic analysis between siControl and siKCNK3 conditions would identify the crucial signaling pathways to better understand the contribution of KCNK3 dysfunction to PAH pathobiology. We used a Liquid Chromatography-Tandem Mass Spectrometry (LC-MS/MS)-based proteomic approach coupled with bioinformatics analysis to understand the consequences of KCNK3 dysfunction in hPASMCs and hPAECs.

## 2. Material and Methods

### 2.1. Human PASMCs and PAECs Cell Culture

Human lung specimens were obtained at the time of a lobectomy or pneumonectomy for localized lung cancer from control subjects. Pulmonary arteries were excised at a distance from tumor areas. hPAECs and hPASMCs were cultured as previously described [10,11], and were used for the study at passage 4. The patients studied were part of a program approved by the institutional Ethics Committee, and had given their written informed consent (ID RCB: 2018-A01252-53, approved on June 18, 2006). Five patients for the condition of hPAECs and 5 patients for hPASMCs were compared in this experiment, and each of these cohorts was duplicated into siControl vs. siKCNK3 conditions, giving a total of 20 samples. The results and statistics were taken from an average of 5 patients per condition.

### 2.2. Reverse Transcription Quantitative PCR (RT-qPCR)

Total RNA was extracted using TRIzol reagent according to standard procedures. One microgram of total RNA was reverse-transcribed using a QuantiTect Reverse Transcription Kit (Qiagen, Valencia, CA, USA; cat. no. 205311). Gene expression was quantified using qPCR following the standard protocol for ready-to-use TaqMan gene expression assays (Thermo Scientific, Villebon-sur-Yvette, France) on a StepOne Plus Real-Time PCR System (Life Technologies, Carlsbad, CA, USA). The predesigned probe sets used for experiments were human KCNK3 (Hs00605529_m1 Thermo Scientific, Paris, France) and human 18S (Hs99999901_s1 Thermo Scientific, Villebon-sur-Yvette, France).

### 2.3. Western Blot Analyses

Total proteins from hPASMCs and hPAECs or rat lung-tissue samples were prepared as described previously [12]. Anti-HO-1 (Abcam; ab13248) was used at 1/1000, anti-IFIT3 (Clinisciences, Nanterre, France; sc-393396) was used at 1/1000, and anti-β-Actin was used for normalization.

### 2.4. Protein Preparation

A total of 20 µg of proteins from each sample was loaded per land on a 12% SDS-PAGE. A short migration was then performed, allowing the proteins to penetrate the gel, and the gels were stained with Coomassie colloidal blue (EZblue, Sigma–Aldrich, Lyon, France) in order to recover a 2 mm band containing all the proteins.

### 2.5. Protein In-Gel Digestion

A reduction/alkylation step was performed with dithiothreitol (DTT) at 10 mM and iodoacetamide at 55 mM. Pieces of the gels were rehydrated at 4 °C in 12 ng/µL of sequencing-grade modified trypsin (Promega, Charbonnières-Les-Bains, France) solubilized in 25 mM of NH_4_HCO_3_ for 1 h and then digested at 37 °C overnight. After tryptic digestion, the peptides were extracted by incubating gel pieces in extraction solvent (0.1% AF/50% ACN).

### 2.6. Liquid Chromatography-Tandem Mass Spectrometry (LC-MS/MS) Analysis

As described in a previous study [13], samples were analyzed on a nano-LC Ultra 2D (eksigent) on-line with a high-resolution mass spectrometer Q-Exactive (Thermo Scientific, France) using a nanoelectrospray interface in positive polarity mode. For each sample, 4 µL of protein digest was loaded onto a Biosphere C18 Precolumn (0.1 × 20 mm, 100 Å, 5 µm; Nanoseparation) at 7.5 µL/min and desalted with 0.1% (*v/v*) formic acid and 2% (*v/v*) Acetonitrile. After 3 min, the precolumn was connected to a Biosphere C18 Nanocolumn (0.075 × 300 mm, 100 Å, 3 µm; Nanoseparation). 

Electrospray ionization was performed at 1.6 kV with an uncoated capillary probe. The buffers were 0.1% formic acid in water (A) and 100% ACN (B). Peptides were separated using a linear gradient from 5% to 30% buffer B for 75 min at 300 nL/min. One run took 95 min, including the regeneration step at 95% buffer B and the equilibration step at 95% buffer A. 

Peptide ions were analyzed using the Xcalibur 4.01 Tune 2.7 (Thermo Scientific, Villebon-sur-Yvette, France) with the following data-dependent acquisition steps: (1) MS scan (mass-to-charge ratio (m/z) of 350 to 1400, 70,000 resolution, profile mode), (2) MS/MS (17,500 resolution, normalized collision energy of 27, profile mode). Step 2 was repeated for the eight major ions detected in step (1). Dynamic exclusion was set to 50 s.

### 2.7. Data Processing and Statistical Analysis

Peak lists were generated as mzXML files using rgw MSConvert software (ProteoWizard 3.0). Mass spectrometric data were searched using X!TandemPipeline software version 0.2.38 developed by the PAPPSO facility [14]. Peptide searches were performed with the following parameters: enzymatic cleavage by trypsin digestion with one possible miscleavage, precursor mass tolerance of ±10 ppm, fragment mass tolerance of 0.5 Da, static modifications of carbamidomethylated cysteine, and potential modification of oxidized methionine.

The Uniprot KB/SwissProt *uniprot_human_organism_9606* database (2019) and a homemade contaminant database (trypsin, keratine, etc.) were used. The identified proteins were filtered with a minimum of two different peptides with a peptide E-value < 0.05 and a protein E-value (product of unique peptide E values) < 10^−5^. Combine analysis mode with all the samples was performed, and the results were collected from the grouping proteins. Within each group, proteins with at least one specific peptide relative to other members of the group were reported as subgroups. One subgroup represents one specific protein. Proteins are characterized with their spectral number. The false discovery rates (FDR) obtained for the peptide and protein identifications with a decoy databank were 0.05% and 0%, respectively.

The label-free comparative quantification of proteins was achieved through spectral counting (SC) and eXtracted Ion Current (XIC) analysis following alignment by Mass ChroQ version 2.2.12 [15].

### 2.8. Statistical Analysis

Statistical analysis was performed using the MassChroqR package developed by the PAPPSO team (R Studio version 1.0.153). After checking the normality and homogeneity of the SC data, proteins showing less than 5 spectra in any of the injections or showing a variation between the minimal and the maximal mean abundance below 1.5 in their number of spectra were removed. For XIC analysis, the peptide intensities measured as peak areas were automatically log10-transformed. Analysis included the following steps: checking the homogeneity of all runs in terms of the chromatography and signal (discarding peptides-mz with an RT > 20 s and chromatographic pics > 200 s) and normalizing the data based on the median. The RT method, keeping only peptides showing a reasonable distribution, eliminating shared peptides, eliminating peptides with more than 10% missing values and imputing missing values for peptides and proteins. By using or not using a filter on the correlation levels of the individual peptides with the mean of their protein (parameters: rCutoff = 0.5, pvalCutoff = 1), two separated lists of proteins have been produced and combined at the end of the XIC analysis. The combination of the XIC and SC analyses allowed retrieving a total of 415 proteins, showing a significant abundance variation (*p* > 0.05) between both conditions.

For Western blot and RT-qPCR analyses, the data were verified for normal distribution using the Shapiro–Wilk test for normality. These values are reported as the mean ± SEM. The difference between the two groups was assessed by a two-tailed unpaired Student’s test. Differences were considered statistically significant at *p-*values < 0.05.

## 3. Results

### 3.1. Validation of KCNK3 Knockdown in hPASMCs and hPAECs

**Figure 1:**
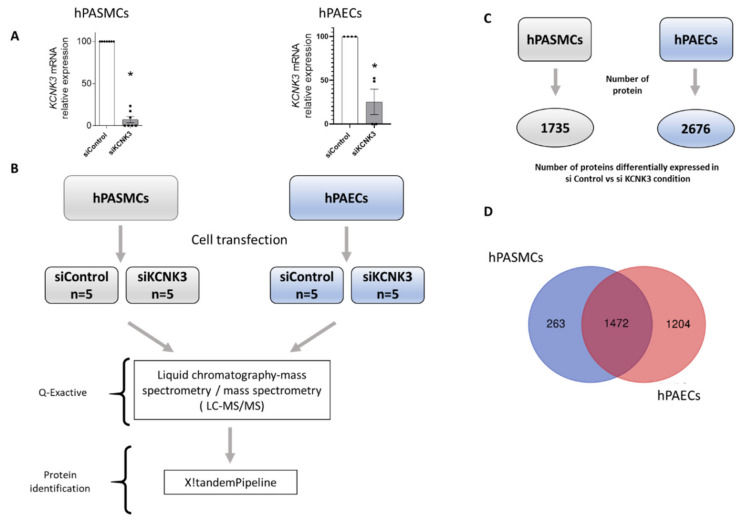
Experimental procedure for the proteomic approach. (**A**) siRNA validation by RT-qPCR in hPASMCs (n = 5) and hPAECs (n = 5). * *p* < 0.05 vs. control (**B**) Experimental process for the LC-MS/MS approach. (**C**) Number of proteins differentially expressed in siControl vs. siKCNK3 in hPASMCs and hPAECs. (**D**) Venn diagram representing the number of proteins identified in hPASMCs only, hPAECs only, and both hPASMCs and hPAECs.

To decipher the changes in the proteome in response to the knockdown of *KCNK3*, an LC-MS/MS-based proteomics approach was carried in hPASMCs and hPAECs. First, to evaluate the molecular consequences of the KCNK3 loss of expression in hPASMCs and hPAECs, we reduced the KCNK3 expression using siRNA against *KCNK3*. As presented in Figure 1A, 72 h after cell transfection, the *KCNK3* mRNA was decreased by 85% in hPASMCs and by 75% in hPAECs (Figure 1A). Then, we used proteomic approach to identify and measure the relative abundance of proteins differentially expressed in hPASMCs or hPAECs transfected with siControl or with siKCNK3 (Figure 1B).

Consistent with the purity of our hPAEC cultures, endothelial specific protein markers were found to be expressed, including Von Willebrand factor, vascular endothelial growth factor receptor 2, platelet endothelial cell adhesion molecule 1, endothelin-converting enzyme 1, angiopoietin-2, and endothelial nitric oxide synthase (not shown).

The purity of hPASMC cultures was confirmed by the absence of specific PAEC markers and the presence of specific smooth muscle markers, including alpha-actinin-1, alpha-actinin-4, myoferlin, actin, alpha cardiac muscle 1, gelsolin, transgelin-2, transgelin, caldesmon, cofilin 2 (muscle) (not shown).

### 3.2. Comparison of Differentially Expressed Proteins Between hPAECs and hPASMCs Treated With siControl or siKCNK3

**Figure 2:**
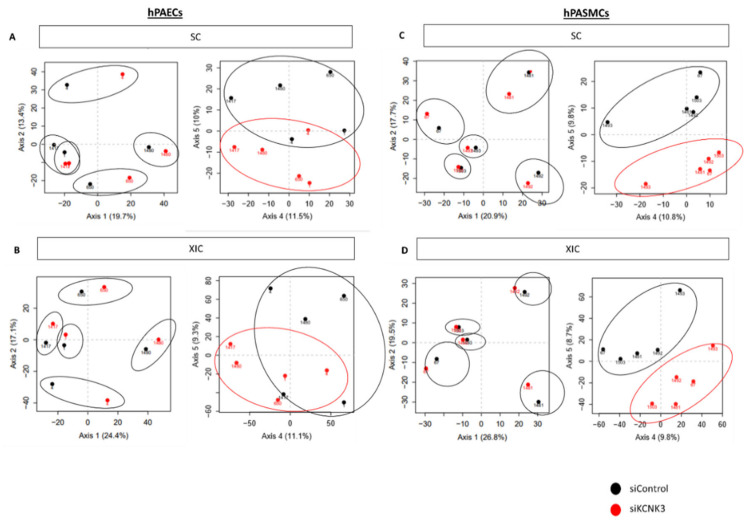
Principal component analysis (PCA) performed on the relative protein expression level. (**A**) PCA showing the consequence of KCNK3 knockdown on the hPAEC proteome obtained by spectral counting (SC) (siControl (black) or siKCNK3 (red)). (**B**) PCA showing the consequence of KCNK3 knockdown on the hPAECs proteome obtained by eXtracted Ion Current (XIC) analysis (siControl (black) or siKCNK3 (red)). (**C**) PCA showing the consequence of KCNK3 knockdown on the hPASMC proteome obtained by SC analysis (siControl (black) or siKCNK3 (red)). (**D**) PCA showing the consequence of KCNK3 knockdown on the hPASMCs proteome obtained by XIC analysis and SC analysis (siControl (black) or siKCNK3 (red)). All are represented with axes 1–2 and axes 4–5.

We identified a total of 2676 proteins differentially expressed in hPAECs transfected with siControl vs. siKCNK3 (Supplementary Table S1), and a total of 1735 proteins differentially expressed in hPASMCs transfected with siControl vs. siKCNK3 (Supplementary Table S2) (Figure 1C). As shown in Figure 1D, 1472 proteins were common in both hPAECs and hPASMCs, 1204 proteins were specific to hPAECs, and 263 were specific to hPASMCs (Figure 1D). Principal component analysis (PCA) based on the relative protein expression levels showed the differences between each patient in terms of hPASMCs and hPAECs (Figure 2A,B and Figure 2C,D, respectively), highlighting the proteome diversity in each patient, represented in axis 1. PCA also revealed the presence of two protein clusters in the hPASMCs and hPAECs experiments by separating the siControl and siKCNK3 samples under axis 4 and 5. These results demonstrated that *KCNK3* knockdown induces proteome modification in hPASMCs and hPAECs.

**Figure 3:**
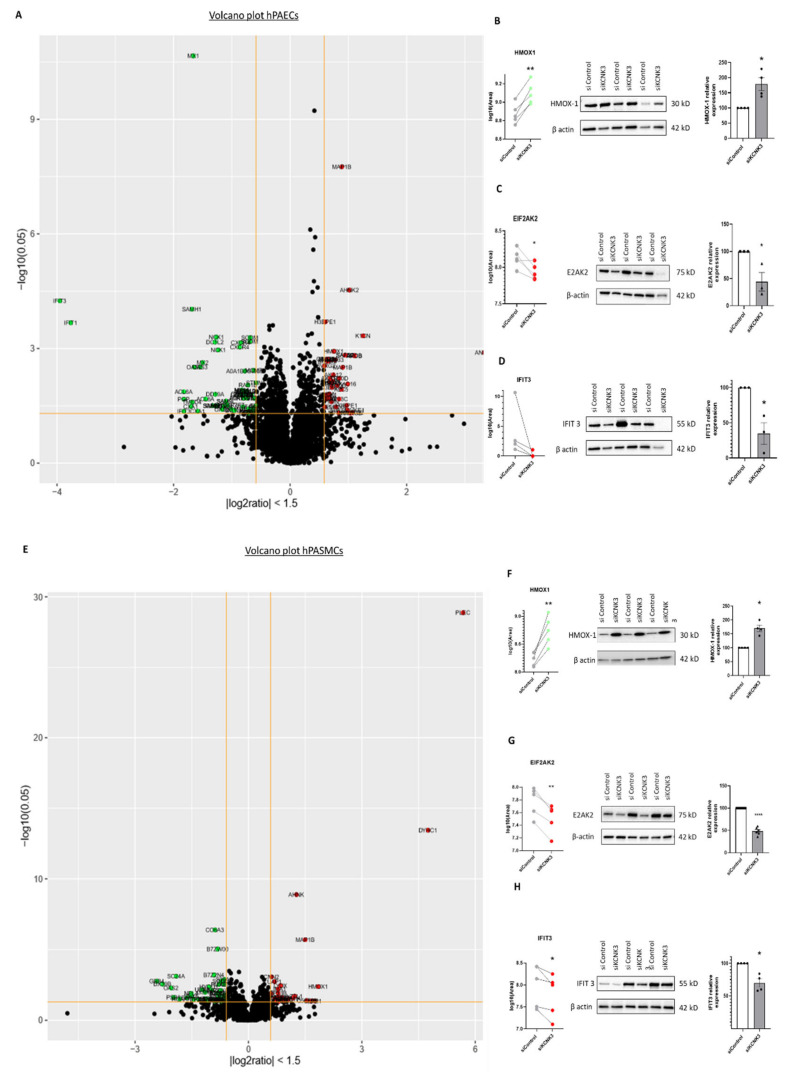
Volcano plot of dysregulated proteins in *KCNK3*-knockdown hPAECs and hPASMCs, and confirmation by Western blot. (**A**) Volcano plot representation of dysregulated proteins in hPAECs transfected with siKCNK3 according to their fold change (outside the yellow line interval fold change > 1.5 or < −1.5) and *p*-value (above the yellow line *p*-value < 0.05). (**B**) Comparison of the Heme Oxygenase-1 (HMOX-1 or HO-1) abundance by LC-MS/MS and Western blot analysis from the same hPAECs lysates. (**C**) Comparison of the EIF2AK2 abundance by LC-MS/MS and Western blot analysis from the same hPAECs lysates. (**D**) Comparison of Interferon-induced protein with tetratricopeptide repeats 3 (IFIT3) abundance by LC-MS/MS and Western blot analysis from the same hPAECs lysates. (**E**) Volcano plot representation of dysregulated proteins in siKCNK3 hPASMCs according to their fold change (outside the yellow line interval fold change > 1.5 or < −1.5) and *p*-value (above the yellow line *p*-value < 0.05). (**F**) Comparison of the HMOX-1 (HO-1) abundance by LC-MS/MS and Western blot analysis from the same hPASMCs lysates. (**G**) Comparison of the Eukaryotic Translation Initiation Factor 2 Alpha Kinase 2 (EIF2AK2 or PKR) abundance by LC-MS/MS and Western blot analysis from the same hPASMCs. (**H**) Comparison of the IFIT3 abundance by LC-MS/MS and Western blot analysis from the same hPASMCs lysates. * *p* < 0.05, ** *p* < 0.01, **** *p* < 0.0001 vs. control. Experiments were analyzed using the Mann–Whitney test.

The distribution of all the differentially expressed proteins in the *KCNK3*-knockdown hPAECs (Figure 3A) and hPASMCs (Figure 3D) were illustrated in a volcano plot. The 10 proteins with the highest fold change in KCNK3-silenced hPAECs were interferon-induced proteins with tetratricopeptide repeats 3 (IFIT3), interferon-induced protein with tetricopeptide repeats 1(IFIT1), phosphoglycolate phosphate (PGP), actine-like protein 6A (ACL6A), gap junction protein alpha-1 (CXA1), SAM and HD domain containing deoxynucleoside triphosphate triphosphohydrolase 1 (SAMH1), transmembrane emp24 protein transport domain containing 4 (TMED4), MX dynamin-like GTPase 1 (MX1), 2′-5′-oligoadenylate synthase 2(OAS2), and 2′-5′-oligoadenylate synthase 3(OAS3) (Supplementary Table S1). In *KCNK3*-silenced hPASMCs, the 10 proteins with the highest fold change were G-protein subunit beta 4 (GBB4), DExD-Box helicase 39B (DX39B), proteasome 20S subunit beta 8 (PSB8), SEC24 Homolog A COPII coat complex component (SC24A), heme oxygenase 1 (HMOX1), LDL receptor-related protein 1 (LRP1), heterogeneous nuclear ribonucleoprotein A/B (ROAA), prosaposin (SAP), cyclase-associated actin cytoskeleton regulatory protein 2 (CAP2), microtubule-associated protein 1B (MAP1B) (Supplementary Table S2).

Among these proteins, we selected HMOX1 (or HO-1) and IFIT3 for confirmation by the semi-quantitative Western blot approach to confirm these findings due to the importance of HMOX1 and EIF2AK2 (or PKR) in the pulmonary field [12] and due to the novelty of IFIT3 in the pulmonary hypertension field.

Among these proteins, the HMOX1/HO-1 abundances’ behaviors were investigated by Western blot. Consistent with the proteomic analysis (Figure 3B,C and Figure 3E,F), the Western blot analyses showed that the HMOX-1 protein expression was significantly increased in the hPAECs (Figure 3B) and hPASMCs (Figure 3F) transfected with siKCNK3 compared to the siControl conditions, in the same way as the proteomic analysis (Figure 3A,B,E,F, Supplementary Tables S2 and S4). We also analyzed the EIF2AK2 protein expression by Western blot in hPAECs and hPASMCs transfected with siKCNK3, and we found a significant decrease in the EIF2AK2 expression in *KCNK3*-knockdown hPAECs (Figure 3C) and hPASMCs (Figure 3G) similar to the proteomic results (Figure 3A,C,E,G). Moreover, we analyzed by Western blot the IFIT3 protein expression, and we found a significant decrease in the IFIT3 expression in *KCNK3*-knockdown hPAECs (Figure 3D) and in hPASMCs (Figure 3H), similar to the proteomic results (Figure 3A,D,E,H). Altogether, these results confirmed our results from the proteomic experiments.

### 3.3. Global Consequences of KCNK3 Knockdown in hPAECs and hPASMCS

**Figure 4:**
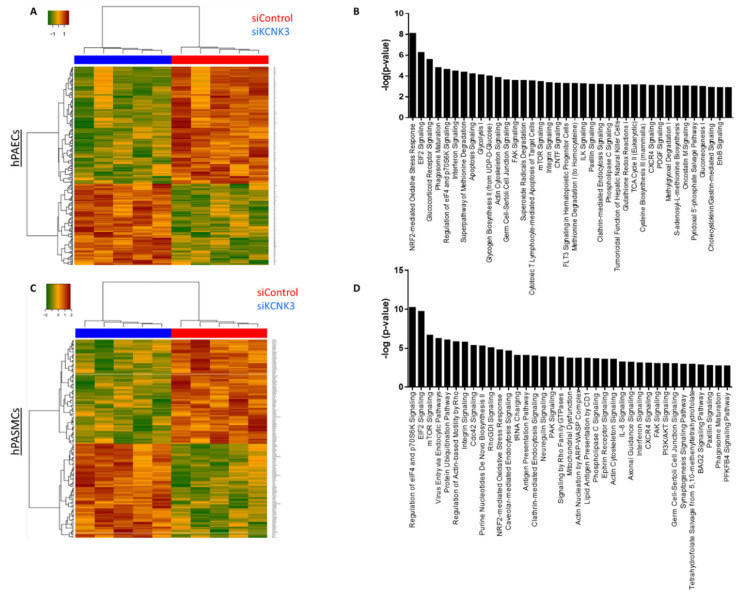
Heatmap of the proteomic results and pathways deregulated in siKCNK3 hPASMCs and hPAECs. (**A**) Heatmap representation for proteomic results in hPAECs. hPAECs from the same patient between the siControl and siKCNK3 conditions are clustering together. (**B**) Representation of the 37 major pathways dysregulated in siKCNK3 hPAECs found with IPA (Ingenuity Pathway Analysis) software. (**C**) Heatmap representation of proteomic results in hPASMCs. hPASMCs from the same patient between the siControl and siKCNK3 conditions are clustering together. (**D**) Representation of the 37 major pathways dysregulated in hPASMCs transfected with siKCNK3 found with the Ingenuity Pathway Analysis (IPA) software.

As highlighted by the heatmap clustering of differentially expressed proteins of each group of conditions (Figure 4A–D, we found that 157 proteins were significantly upregulated in siKCNK3, while 247 proteins were significantly downregulated in siKCNK3-transfected hPAECs (Figure 4A–D and Supplementary Table S3). A total of 111 proteins were significantly upregulated in siKCNK3, while 154 proteins were significantly downregulated in siKCNK3-transfected hPASMCs (Figure 4C,D and Supplementary Table S3). To identify the canonical pathways, predicted upstream regulators, biological functions, and disease, the significant proteins differentially expressed were analyzed using Ingenuity Pathway Analysis (IPA, Ingenuity Systems, www.ingenuity.com). The IPA analysis revealed differences in 30 canonical pathways (z-score > 1 or −1), 48 upstream regulators, 15 biological functions, and 20 disease and functional networks between KCNK3-knockdown hPAECs. In the hPASMCs, the IPA analysis found significant differences in 14 canonical pathways (z score > 1 or −1), 16 upstream regulators, 9 biological functions, and 13 disease and functional networks. The activation Z-score makes predictions about potential regulators by using information about the direction of gene regulation [16].

### 3.4. Biological Consequences of KCNK3 Knockdown in hPAECs

To identify the most interesting signaling pathways, networks, diseases, and biological functions deregulated by the loss of KCNK3 expression, we separated the hPAECs specific proteome (316 proteins), the hPASMCs specific proteome (222 proteins), and the common proteome of both hPAECs and hPASMCs (53 proteins) (Supplementary Table S3).

For the hPAECs-specific proteome, we selected the five most affected canonical pathways by the combination of the highest −log(*p*-value) in association with the highest z-score. We used the IPA regulation z-score algorithm to identify the canonical pathways, upstream (positive z-score) or downstream (negative z-score) regulators, and biological functions according to our proteomic data. (positive z-score) according to our RNAseq data. In order to enhance the stringency of our analysis, we considered only functions with a z-score < −1.5 or > 1.5. Among them, our results indicate that Eukaryotic Initiation Factor 2 (EIF2) signaling (z score −1.89) was reduced in the *KCNK3-*knockdown hPAECs, and the mammalian target of rapamycin (mTOR) signaling (z-score 2.23) and superpathway of methionine degradation (z-score 2.82) were activated in the *KCNK3*-knockdown hPAECs (Supplementary Table S4a). EIF2 signaling integrates diverse stress-related pathways to regulate both the global and specific mRNA translation. mTOR signaling is known to be a master of growth regulator that senses and integrates diverse nutritional and environmental alterations, while the superpathway of methionine degradation, as indicated by the name, describes the degradation of the amino acid L-methionine in mammals. 

**Figure 5:**
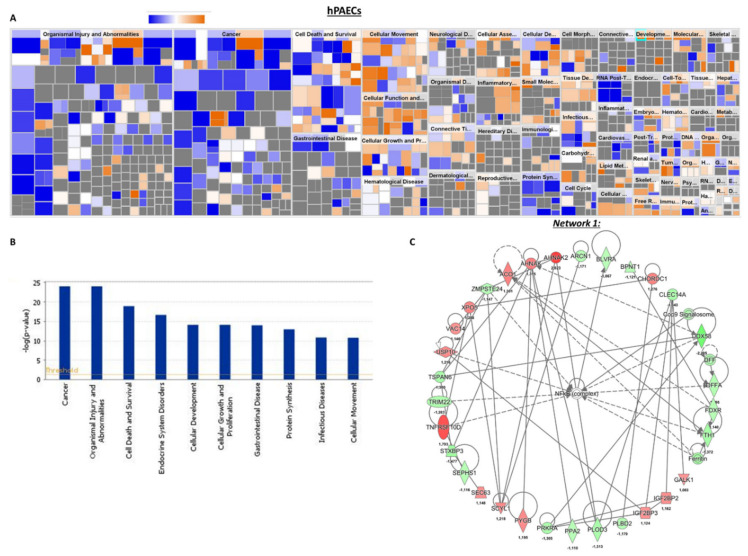
Deregulated biological functions in *KCNK3*-knockdown hPAECs and the protein network of interactions. (**A**) Heatmap representing the up-regulated (orange) or down-regulated (blue) biological functions in hPAECs transfected with siKCNK3. (**B**) Histogram representing the 10 first deregulated biological functions in hPAECs transfected with siKCNK3, classified according to –log (*p*-value) (**B**). (**C**) Representation of the dysregulated network 1: protein synthesis, molecular transport, hereditary disorder. Red proteins are up-regulated proteins and green proteins are down-regulated proteins.

In association with deregulated pathways, IPA identified abnormal biological functions such as the splicing of RNA, the processing of mRNA, neuronal cell death, cell survival, the proliferation of EC lines, neoplasia, and the catabolism of protein was found to be inhibited in *KCNK3*-knockdown hPAECs (Figure 5A,B and Supplementary Table S4b), while cell death, the metabolism of membrane lipid derivative, and the cell proliferation of breast cell line were found to be activated in *KCNK3* knockdown-hPAECs (Figure 5A,B and Supplementary Table S4b).

Moreover, the IPA identified in *KCNK3*-knockdown hPAECs six upstream molecules that should be activated, including lysine demethylase 5 (KDM5), histone desacetylase 2 (HDAC2), transcription factor AP-2 alpha (TFAP2A), and NK2 Homebox 3 (NKX2-3). IPA also identified seven upstream molecules should be inhibited, including interferon alpha, interferon alpha 2 (IFNA2), serine/threonine kinase 11 (STK11), and interferon gamma (IFNG) (Supplementary Table S4c). 

The differentially expressed proteins in *KCNK3*-knockdown hPAECs were associated with 19 functional networks, including protein synthesis, molecular transport (network 1) and RNA post-transcriptional modification, and cancer (network 2) (Figure 5C and Supplementary Table S4d). In this network, we found, for example, that ferritin, ferredoxin reductase (FDXR), and ferritin heavy chain 1 (FTH1) were downregulated, while TNF Receptor Superfamily Member 10d (TNFRSF10D) was upregulated (Figure 5C). These results indicate that the cellular iron storage was reduced in the absence of KCNK3, as well as TNFRS10D signaling, which is related to p53 and ERK signaling.

### 3.5. Biological Consequences of KCNK3 Knockdown in hPASMCs

In hPASMCs, our results indicated that four canonical pathways were reduced in *KCNK3*-knockdown hPASMCs: EIF2 signaling (z-score = °2), Purine Nucleotides De Novo Biosynthesis II (z-Score= −2), and Interleukine-8 (IL-8) signaling (z-score = −2.23), while one canonical pathway was activated: phosphoinositide 3-kinase/serine/threonine kinase Akt (PI3K/AKT) signaling (z-score = 2.45) (Supplementary Table S5a). These results suggest an activation of the PI3K/AKT pathway and an inhibition of EIF2, Purine Nucleotides De Novo Biosynthesis II, and IL-8 pathways in *KCNK3*-knockdown hPASMCs. Purine Nucleotides De Novo Biosynthesis II is highly conserved among organisms, participating in many aspects of cellular metabolism, including the structure of DNA and RNA, serving as an enzyme cofactor, functioning in cellular signaling, acting as a phosphate group donor, and generating cellular energy. IL-8 is a pro-inflammatory CXC chemokine which is described to regulate gene expression via numerous transcription factors, modulate the cellular proteome by acting at the translational level, and modulate the cytoskeleton cell organization by acting at the posttranslational level. The PI3K/AKT pathway acts as an important regulator of the cell cycle that is directly related to cellular quiescence, proliferation, and cancer, and is described to be over-activated in PAH.

**Figure 6:**
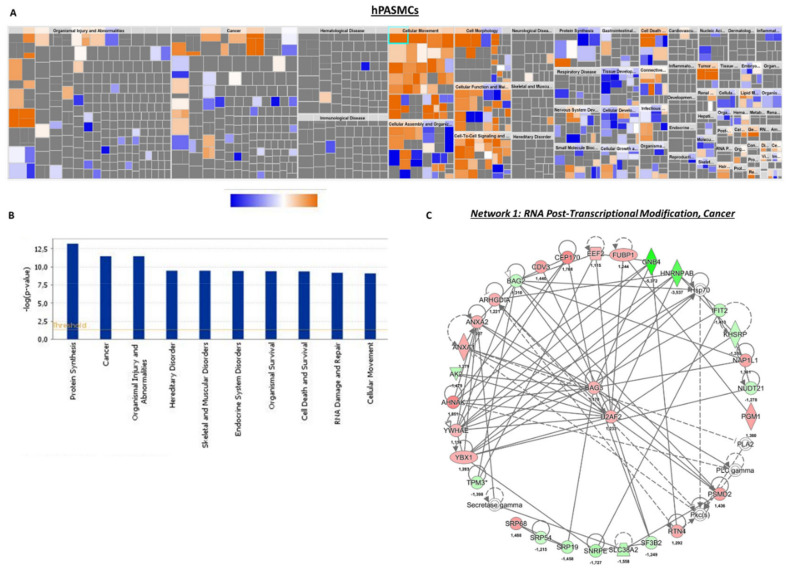
Deregulated biological functions in *KCNK3*-knockdown hPASMCs and the protein network of interactions. (**A**) Heatmap representing the up-regulated (orange) or down-regulated (blue) biological functions in hPASMCs transfected with siKCNK3. (**B**) Histogram representing the 10 first deregulated biological functions in hPASMCs transfected with siKCNK3, classified according to –log (*p*-value). (**C**) Representation of the dysregulated network 1: RNA post-transcriptional modifications, cancer, and hematological disease. Red proteins are up-regulated proteins and green proteins are down-regulated proteins.

Underlying these alterations, the IPA predicted that four upstream molecules should be activated among them: ten-eleven-translocation 2 (TET2), lysine demethylase 5A (KDM5A), and homeobox A10 (HOXA10). The IPA predicted that seven upstream molecules should be inhibited in *KCNK3*-knockdown hPASMCs, among them MLX Interacting Protein Like (MLXIPL) and Heat Shock Protein 90 Beta Family Member 1 (HSP90B1) (Supplementary Table S5b). In association with these deregulated pathways, the IPA identified abnormal biological functions, such as the cell spreading and shape change of tumor cell lines, and cell movement, cell attachment, and cell death were found to be significantly activated in *KCNK3*-knockdown hPASMCs (Figure 6A,B and Supplementary Table S5c).

The differentially expressed proteins in *KCNK3*-knockdown hPASMCs were associated with 12 functional networks, including RNA post-transcriptional modification, cancer, hematological disease (network 1), RNA damage and repair, protein synthesis, gene expression (network 2) (Figure 6C and Supplementary Table S5d). In this network, we found, for example, that Guanine Nucleotide-Binding protein subunit beta-4 (GNB4) was downregulated, while Centrosomal Protein 170 (CEP170) and (AHNAK) were upregulated (Figure 5C). The reduced expression of GNB4 could have as a consequence a reduced GTPase activity in *KCNK3*-knockdown hPASMCs, while the over-expression of CEP170 and AHNAk should contribute to the enhancement of microtubule organization, cell structure and migration, and calcium channel regulation in *KCNK3*-knockdown hPASMCS. 

### 3.6. Common Deregulated Pathways to KCNK3-Knockdown hPAECs and hPASMCs

**Figure 7:**
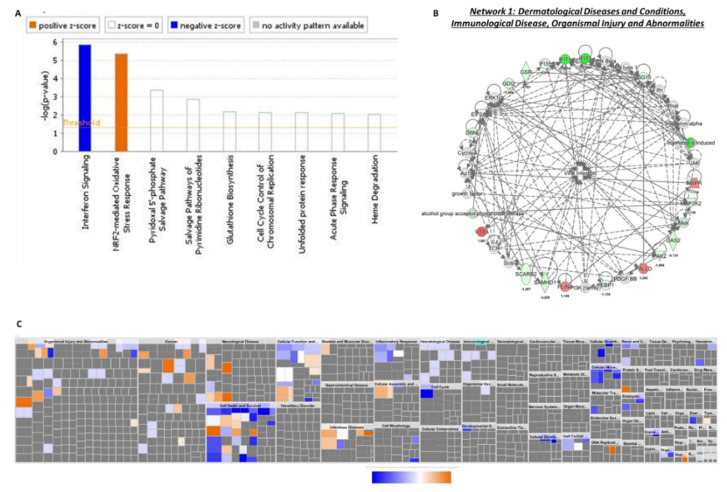
Common deregulated functions in hPASMCs and hPAECs transfected with siKCNK3. (**A**) Histogram representing the 2 deregulated canonical pathways in hPAECs and hPASMCs transfected with siKCNK3, using the fold change obtained in hPAECs transfected cells. (**B**) Heatmap representing the up-regulated (orange) or down-regulated (blue) biological functions in hPAECs and hPASMCs transfected with siKCNK3, using the fold change obtained in hPAECs transfected cells. (**C**) Representation of dysregulated network 1: immunological disease and organismal injury and abnormalities. Red proteins are up-regulated proteins and green proteins are down-regulated proteins.

**Figure 8:**
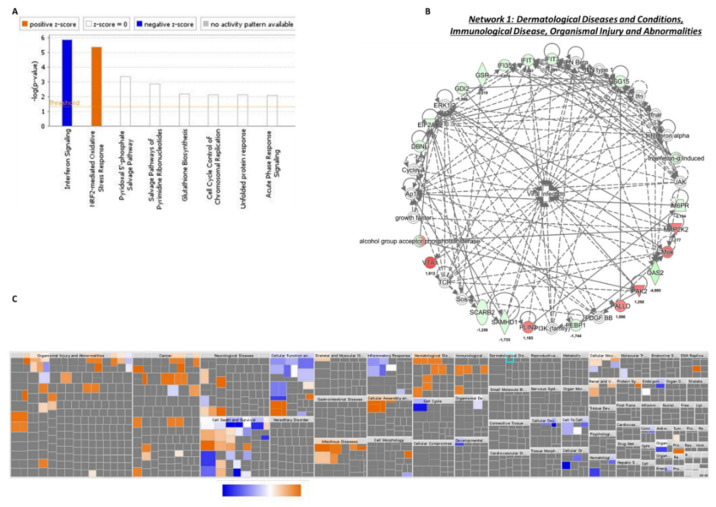
Common deregulated functions in hPASMCs and hPAECs transfected with siKCNK3. (**A**) Histogram representing the 2 deregulated canonical pathways in the hPAECs and hPASMCs transfected with siKCNK3, using the fold change obtained in hPASMCs transfected cells. (**B**) Heatmap representing the up-regulated (orange) or down-regulated (blue) biological functions in hPAECs and hPASMCs transfected with siKCNK3, using the fold change obtained in hPASMs transfected cells. (**C**) Representation of dysregulated network 1: immunological disease and organismal injury and abnormalities. Red proteins are up-regulated proteins and green proteins are down-regulated proteins.

We found that 53 proteins were common to hPAECs and hPASMCs transfected with siKCNK3 (Supplementary Table S6a). Using IPA, we identified two canonical pathways with a z-score value, NRF2 (nuclear factor erythroid 2-related factor 2) and interferon signaling, with s z-score prediction of 1.34 and −2, respectively, in hPASMCs, and with a z-score predicted value of 0.44 and −2, respectively, in hPAECs (Figure 7A and Figure 8A). These results suggest an activation of NRF2 pathway and an inhibition of interferon signaling in *KCNK3*-knockdown hPAECs and hPASMCs. NRF2 is a transcription factor that regulates several genes (>600) involved in glutathione synthesis, antioxidant proteins, enzyme activity, transporter activity, metabolism, and transcription. The interferon signaling plays a critical role in the immune response, conferring a cellular resistance to viral infection.

Based on our enrichment data, the IPA identified that 10 molecules could explain the decrease in interferon signaling. These inhibited upstream molecules were interferon lambda 1 (IFNL1), interferon regulatory factor 1 (IRF1), interferon beta (IFN Beta), interferon alpha 2 (IFNA2), interferon alpha/beta receptor (IFNAR), interferon regulatory factor 7 (IRF7), interferon gamma IFNG, interferon regulatory factor 3 (IRF3), interferon beta 1(IFNB1), and interferon alpha (Supplementary Table S6b). Moreover, the IPA predicted that only four molecules were activated: bruton tyrosine kinase (BTK), atypical chemokine receptor 1 (ACKR2), mitogen-activated protein kinase 1 (MAPK1), and ETS variant transcription facto 6-RUNX family transcription factor 1 (ETV6-RUNX1) (Supplementary Table S6b). IPA identified that these dysregulated pathways were related to abnormal biological functions, such as the cell death of myeloid cells, and cell cycle progression was found to be significantly inhibited, while DNA damage was found to be significantly activated in both hPAECs and hPASMCs lacking KCNK3 (Figure 7B and Figure 8B and Supplementary Table S6c).

Finally, the common differentially expressed proteins in *KCNK3*-knockdown hPAECs and hPASMCs were associated with four functional networks, including the networks immunological disease, organismal injury, and abnormalities (network 1) (Figure 7C and Figure 8C, and Supplementary Table S6d). In this network and in accordance with the previous upstream identified molecules, we found, for example, that interferon induced protein 35 (IFI35), IFIT1, IFIT3, and interferon-α induced proteins were downregulated in hPAECs and hPASMCs transfected with siKCNK3, as well as Eukaryotic Translation Initiation Factor 2 Alpha Kinase 2 (EIF2AK2) (Figure 7C and Figure 8C). Common to hPAECs and hPAECs, vesicle trafficking 1 (VTA1) was overexpressed in siKCNK3 conditions (Figure 7C and Figure 8C). The reduced expression of interferon-signaling proteins suggest that, in the absence of KCNK3, hPASMCs and hPAECs should have a drop in their viral infection response. Whereas, the overexpression of VTA1 should contribute to the intensification of lysosomal protein degradation in *KCNK3*-knockdown hPAECs and hPASMCS. These common deregulated pathways in hPAECs and hPASMCs have highlighted the fact that KCNK3 has an unexpected similar function in hPAECs and hPASMCs, despite the different physiological cell functions, suggesting that these commonly identified pathways are directly related to KCNK3 and independent of cell systems.

## 4. Discussion

KCNK3 reduced function and expression is a hallmark of PAH. KCNK3 dysfunction is described to lead to the plasma membrane depolarization of PASMCs, pulmonary artery vasoconstriction, and increased PASMC proliferation. However, the molecular mechanisms linked to KCNK3 dysfunction in hPASMCs and hPAECs are mostly unknown. 

In the present study, beyond the deregulated pattern expected in *KCNK3* knockdown in hPASMCs and hPAECs, the proteomic approach has highlighted new relevant deregulated pathways. Indeed, the KCNK3 loss of expression induces the inhibition of interferon signaling, EIF2 signaling (in hPAECs and hPASMCs), and purine nucleotides de novo biosynthesis II and IL-8 signaling (in hPASMCs). In addition, KCNK3 loss of expression induces the activation of the NRF2 pathway (in hPAECs and hPASMCs), glycolysis, apoptosis signaling, the superpathway of methionine degradation and mTOR signaling (in hPAECs), and PI3K/AKT signaling (in hPASMCs). These results help us to better understand the pathophysiological consequences of KCNK3 dysfunction in pulmonary vascular cells and their implications in PAH pathogenesis. In PAH, PAECs dysfunction is characterized by the impairment of endothelial-dependent vasodilatation, by a reduction in anticoagulant properties, by metabolic changes, by the production of reactive oxygen species, and by an unadapted release of pro-inflammatory molecules. These dysfunctions result in PAECs phenotypic changes, including the decreased capacity for vascular tube formation, increased aerobic glycolysis, the loss of several endothelial markers, the acquisition of several mesenchymal markers and a pro-inflammatory phenotype, and increased *in vitro* hPAECs proliferation [9]. Consequently to PAEC dysfunction, hPASMCs are deregulated and characterized by enhanced proliferation, survival, vasoconstriction, and cell movement in PAH [9].

We recently found that *KCNK3* knockdown is associated with ERK-1/2 activation, HIF1α stabilization, and the over-phosphorylation of AKT, promoting the proliferation of control hPASMCs [8]. Here, with our analysis we found that *Kcnk3* deficiency is in favor of cell spreading, cell movement, and cell invasion, as well as some canonical pathways linked to cell proliferation (mTOR, PI3K/AKT) and DNA damage. These functions have been well described to promote cancer and tumor development [17]. Based on numerous points of similarity, PAH has emerged as a cancer-like disease, and is characterized by abnormal cell proliferation, aberrant resistance to apoptosis, and the deregulation of cellular metabolism [17], providing new concepts and new therapeutic perspectives for PAH. A large spectrum of growth factors, cytokines, and chemokines has been demonstrated to promote or to initiate PAH and cancer, including platelet-derived growth factor (PDGF) and interleukin 6 (IL-6) [18]. We found that the knockdown of KCNK3 promotes the proliferation of control hPASMCs [8] 

In the absence of KCNK3, the control hPAECs proliferation rate was unchanged compared to the siControl conditions (not shown); however, the consequence of the KCNK3 loss of expression in the phenotype of hPAECs is unknown. 

### 4.1. HO-1 Relevance in PAH

Common to hPASMCs and hPAECs, we found that *KCNK3* knockdown deregulated the canonical pathway, EIF2 (eukaryotic initiation factor 2) signaling, and eIF4 and p70S6K signaling. EIF2 is crucial for protein synthesis and the initiator binding of tRNA to the ribosome. EIF2 signaling is known to play a critical role in the vascular remodeling and proliferation of PASMCs in chronic hypoxia-induced pulmonary hypertension [19], as well as in pulmonary vein occlusive disease [12]. EIF2 signaling is a part of the integrated stress response (ISR). To restore cellular homeostasis, eukaryotic cells activate a common adaptive pathway in response to stress stimuli. ISR is mediated by several ISR kinases (GCN2, PERK, HRI), and the protein kinase R (PKR)—also named EIF2AK2—is known to phosphorylate the α-subunit of eIF2α, leading to the activation of activating transcription factor 4 (ATF4). ATF4 is known to induce the expression of other transcription factors, including HO-1 [12]. Here, we found that EIF2AK2 is reduced in hPASMCs and hPAECs transfected with siKCNK3. Additionally, in hPASMCs we found that the co-effector of GCN2 (general control nonderepressible 2, another ISR kinase), GCN1, is upregulated in the absence of KCNK3 (Supplementary Table S2). GCN2/GCN1 senses amino acid deficiency and modulates amino acid metabolism as a response to nutrient deprivation [20]. Heme oxygenase-1 (HO-1) is a stress-inducible enzyme HO-1 which maintains cellular homeostasis under stress conditions [21]. HO-1 is well established to play a crucial role in the cell adaptive response to oxidative and cellular stresses, increasing cell proliferation and cell angiogenesis and reducing inflammation and apoptosis [22]. Here, we found that HO-1 was overexpressed in hPASMCs and hPAECs transfected with siKCNK3. HO-1 plays a crucial role in the survival of vascular smooth muscle [23], and could play major role in many human diseases, including pulmonary veino-occlusive disease [12,22]. HO-1 promotes cancer development, since HO-1 overexpression is observed in different types of cancers promoting cell survival [24], while the inhibition of HO-1 reduces cell viability [25]. HO-1 is also described to regulate endothelial-to-mesenchymal transition [26], which is described to contribute to PH pathobiology [27] in relation to *Kcnk3* deficiency in rats [8]. In vascular smooth muscle cells, Liu et al. found that ER stress mediates HO-1 overexpression via the antioxidant response element (ARE), promoting the suppression of apoptosis [28]. NRF2 is the major transcription factor that promotes the ARE pathway [29], and here we found that the NRF2-mediated oxidative stress response is deregulated in *KCNK3*-knockdown hPAECs and hPASMCs, indicating that the loss of KCNK3 is a key signal to activate this pathway and promote cell survival. We also found that *KCNK3* knockdown leads to a reduced EIF2AK2 expression and the overexpression of HO-1, which should lead to increased biliverdin and bilirubin. An elevated serum bilirubin content is found to be a risk factor for death in PAH patients [30]. Moreover, HO-1 activity cleaves the heme to form equimolar ferrous ions, biliverdin-Ixα, and carbon monoxide (CO) [31,32,33]. The toxic properties of CO are well known in pulmonary medicine; CO replaces O2 from hemoglobin, causing tissue hypoxia [34]. At high CO concentrations, there are other mechanisms of CO-induced toxicity, including apoptosis and lipid peroxidation [35]; however, at low concentrations, CO contributes to several physiological reactions. Indeed, CO activates soluble guanylate cyclase (sGC), inducing the production of cyclic 3′:5′-guanosine monophosphate (cGMP) [36].

The sGC/cGMP pathway mediates the effects of CO on smooth muscle cell relaxation [36,37]. CO-induced vascular relaxation could be directly mediated by the activation of calcium-dependent potassium channels (BKCa) [38,39], which could be a compensatory mechanism in the context of KCNK3 dysfunction.

### 4.2. Interferon Signaling Relevance in PAH

The interferon-induced protein with tetratricopeptide repeats (IFIT) gene family has been well studied for their antiviral properties [40]. In most cell types without viral infection, IFIT gene expression is involved in several biological processes, such as virus-induced translation initiation, replication, and double-stranded RNA signaling [41]. The expression of IFIT genes is enhanced by interferon treatment and viral infection [42]. In this study, we found by the LS/MS approach that the IFIT protein family (IFIT1, IFIT3, IFIT5, IFITX) is reduced in hPASMCs and hPAECs transfected with siKCNK3, and by a larger extent in hPAECs. Additionally, we confirmed the reduction in IFIT3 expression by Western blot experiments, indicating that KCNK3 dysfunction leads to IFIT family down-expression. IFIT genes are involved in the response to viral infection as well as KCNK3. In fact, the overexpression of KCNK3 reduces the ability of the HIV-1 accessory protein (Vpu) to release viral particles, while Vpu is described to abolish the TASK-1 current [43]. HIV-1 exploits the host miRNA cellular systems reduce to block the mechanism of innate inhibition. HIV-1 regulates miR-21, which is known to downregulate the KCNK3 expression, allowing a stronger infection efficiency [44]. HIV infection confers an increased risk for a variety of infectious lung diseases, and HIV infection is an established risk factor for PH. The prevalence of PAH is several-fold higher in HIV-infected patients compared with the PH population [45]. Our results suggest that the loss of function of KCNK3 could facilitate PAH in HIV-infected patients.

Due to their antiviral, immunomodulatory, and antitumor properties, interferon type I (IFN1) is naturally overexpressed in the human body in response to pathogens and in tumor cells. We found in a PAH lung increased interferon receptor 1 (IFNAR1) protein levels. We also found that *Ifnar1*^−/−^ mice were protected against the development of PH [46], indicating that IFN1 contributes to PAH pathogenesis. The benefits of IFN therapy in combination with chemotherapy and radiotherapy is well documented for several types of cancers. However, it was also reported that IFN therapy has several side effects, triggering PAH [46,47].

### 4.3. KCNK3 Dysfunction and Superpathway of Methionine Degradation

Here, we found an increase in glycolysis and a decrease in the superpathway of methionine degradation in *KCNK3*-knockdown hPAECs, indicating that the loss of expression of KNCK3 facilitates the glycolysis metabolic shift of hPAECs. In most cancers, enzymes responsible for the Methionine (Met) cycle are altered [48]. Met is also crucial for RNA and protein synthesis, and polyamine [49]. Polyamines increase cell proliferation by reducing cell apoptosis [50].

The effect of Met restriction leads to the inhibition of IGF signaling and mTOR, and a reduction in mitochondrial oxidative stress [51]. In the present study, we found that the superpathway of Met degradation is reduced in hPAECs transfected with siKCNK3, suggesting that the loss of KCNK3 increases the pool of Met by reducing their degradation pathway, which could lead to the activation of the mTOR signaling pathway. Indeed, we also found an activation of the mTOR signaling pathway in *KCNK3*-knockdown hPAECs. The overactivation of the mTOR pathway is well known to contribute to PAH pathogenesis, and the in vivo inhibition of mTOR by rapamycin reduces the development experimental PH [52]. Moreover, mTOR is a part of the PI3K/AKT/mTOR pathway, which we found to be increased in lungs from *Kcnk3*-mutated rats as well as in human PAH [8,10].

Patients with hypermethioninemia exhibit a lot of symptoms, including neurological problems, sluggishness, muscle weakness, liver problems, and unusual breath [53]. A high level of Met could hypermethylate DNA and then produce undesirable epigenetic effects. DNA methylation contributes to the pathogenesis of pulmonary diseases, including cancer and PAH [54], in particular by promoting phenotypic changes in hPAECs in PAH [55]. Alterations in the DNA methylation of the *SOD2* gene (Superoxyde dismutase 2) are known to reduce the expression of SOD2, which is related to pulmonary vascular cell proliferation, apoptosis resistance, and cell metabolic changes [56]. Here, common to hPAECs and hPASMCs, we found that KCNK3 knockdown reduces the expression of SOD2, supporting the role of KCNK3 in promoting PAH via aberrant DNA methylation.

Moreover, in hPAECs (but not in hPASMCs) transfected with siKCNK3, we found a decrease in the expression of gamma-glutamyl hydrolase (GGH). GGH is involved in the turnover of cellular folates, since a reduction in the GGH expression increases the cellular folate content and poly-gamma glutamate. In lung adenocarcinoma, the GGH expression was lower and correlated with higher folate concentrations and increased DNA methylation [57]. In the context of KCNK3 dysfunction and PAH, a downregulation of GGH may have significant implications for the pulmonary vascular cell phenotype.

### 4.4. KCNK3 Dysfunction and Cell Metabolism Changes

In siKCNK3 transfected hPAECs and hPASMCs, we found by the LC/MS approach an activation of glycolysis. PAH pulmonary vascular cells and cancer cells have developed some similar metabolic adaptations [18]. Several studies have shown an increase in glycolysis and a decrease in glucose oxidation in pulmonary vascular cells, similar to the metabolic changes described in proliferating tumor cells [18]. These metabolic changes include the pentose phosphate pathway, glutaminolysis, and fatty acid synthesis and oxidation. In PAH pulmonary vascular cells and in cancer cells, this metabolic shift is mostly driven by HIFs overactivation [18]. Additionally, we recently demonstrated that KCNK3 dysfunction in human and rat PASMCs induces the overexpression of HIF1α [8]. 

Moreover, we found that *KCNK3* knockdown in hPASMCs induced a significant reduction in the glucose-6-phosphate dehydrogenase (G6PD) expression, which is the main producer of NADPH. Clinical reports connect G6PD deficiency with PAH [58]. More, three patients with missense mutations in the G2PD gene were identified in a cohort of PAH patients [59]. Altogether, these reports indicate that G6PD dysfunction could predispose one to the development of PAH. Unexpectedly, an increase in G6PD activity is reported in chronic hypoxia rats [60]. The loss of G6PD was shown to prevent the production of NADPH and a subsequent decrease in NO synthesis, since reduced NADPH is a critical substrate for NO synthesis [61]. These data suggest that the inhibition of G6PD could be deleterious to the PA tone in PH. This is consistent with our recent results, showing that *Kcnk3* deficiency in rats is associated with significant alteration in PA tone, including the impairment of NO-induced relaxation [8].

Limitations: Due to the low capability of mass spectrometry to identify hydrophobic channel proteins, which are particularly nonpolar and present in a very limited amount, unfortunately we did not detect a large amount of ion channel families’ proteins. However, 33 ion channels or transporters in hPAECs and 28 in hPASMCs (data not shown) were identified without any changes in their expression in the absence of KCNK3. 

In the future, the expression of other newly identified signaling pathways will be further investigated by Western blot or PCR analysis in the context of the KCNK3 loss of expression as well as in the more general context of PAH.

In summary, the current study analyses the molecular functional consequences of KCNK3 dysfunction in hPASMCs and hPAECs. Altogether, these findings allow us to better understand the involvement of KCNK3 dysfunction in PAH disease.

## Supplementary Materials

The following are available online at https://www.mdpi.com/1422-0067/21/19/7400/s1.
